# Investigating the Wound-Healing Potential of a Nanoemulsion–Gel Formulation of *Pituranthos tortuosus* Essential Oil

**DOI:** 10.3390/gels10030155

**Published:** 2024-02-20

**Authors:** Badr Bahloul, Enis Ben Bnina, Assia Hamdi, Luis Castillo Henríquez, Dhaou Baccar, Nesrine Kalboussi, Aïmen Abbassi, Nathalie Mignet, Guido Flamini, José Roberto Vega-Baudrit

**Affiliations:** 1 Drug Development Laboratory LR12ES09, Faculty of Pharmacy, University of Monastir, Monastir 5000, Tunisia; hamdiessia@gmail.com (A.H.); dhaou.bak@gmail.com (D.B.); kalboussi.nessrine@gmail.com (N.K.); 2 LR21AGR03-Production and Protection for a Sustainable Horticulture (2PHD), Regional Research Centre on Horticulture and Organic Agriculture, IRESA, University of Sousse, Chott Mariem 4042, Tunisia; benbninae@gmail.com; 3 Chemical and Biological Technologies for Health Group (UTCBS), Université Paris Cité, 75006 Paris, France; luis.castillo-henriquez@etu.u-paris.fr (L.C.H.); nathalie.mignet@u-paris.fr (N.M.); 4 Research Unit “Natural Bioactive Substances and Biotechnology” UR17ES49, Pharmacognosy Laboratory, College of Pharmacy of Monastir, University of Monastir, Monastir 5000, Tunisia; abbassi.aimen@gmail.com; 5 Dipartimento di Farmacia, University of Pisa, Via Bonanno 6, 56126 Pisa, Italy; guido.flamini@unipi.it; 6 National Nanotechnology Laboratory (LANOTEC), National Center for High Technology (CeNAT), San José 1174-1200, Costa Rica; jvegab@gmail.com

**Keywords:** GC-MS, topical drug delivery systems, nanotechnology, nanoemulgel, *Pituranthos tortuosus*, medicinal plants

## Abstract

This study explores a nanoemulsion (NE)-based gel incorporating Tunisian *Pituranthos tortuosus* essential oil, with a focus on its wound-healing potential. The essential oil, extracted via hydrodistillation, underwent GC-MS analysis for compositional verification. The physicochemical characterization included dynamic light scattering (DLS), transmission electron microscopy (TEM), zeta potential measurement, pH, and viscosity. The gelification of the NE facilitated topical application. The results revealed an average extraction yield of 0.45% and identified 38 compounds in the essential oil. The NE exhibited a particle size of 27 ± 0.4 nm, a polydispersity index (PDI) of 0.3, and a zeta potential of −22.8 ± 1.4 mV. The stability of the gelified preparation was confirmed through thermodynamic stability studies, TEM observations, and zeta and size results. In vivo experiments confirmed significant wound-healing effects, highlighting the promising role of the NE-based gel in healthcare advancements. This research underscores the potential of novel phyto-based delivery systems in wound care.

## 1. Introduction

Skin covers the entire human body; it is the largest organ, with a surface of 2 m^2^, and weighs up to 5 kg. It is one of the most complex organs. It plays a defensive role, regulates temperature, preserves hydration, ensures sensory perception, and maintains humeral equilibrium [1].

The skin surface can be damaged with a loss of functionality and integrity, leading to infections [2]. The inflamed and infected wound endangers social, economic, and moral health, such as mandatory absences from work and psychosocial changes that reduce the quality of life and the need for long-term treatments that can be, in some cases, expensive and complex [3]. 

The inability to heal a wound has become a global problem. Reduced drug activity mainly causes this limitation in current devices and techniques, as well as multi-resistant bacteria. Thus, it is necessary to adopt the most effective treatment for patients suffering from infected lesions while preserving a rapid and total skin recovery [4]. Clinicians require deeper knowledge to discover new drugs and find other desirable approaches.

Recently, research has been focusing on biocompatibility, ensuring the inhibition of bacterial growth, moisturizing the wound [5], reducing infection [6], minimizing pain [7], stimulating healing mechanisms [8], accelerating wound closure [9], and reducing scar formation [7]. Healing processes include four overlapping yet coordinated successive stages: hemostasis [10], inflammation [11], re-epithelialization [12], and remodeling [13].

To overcome these drawbacks and develop low-cost approaches to wound healing, researchers have turned to plants to exploit bioactive molecules to treat damaged skin [14,15]. 

Traditional wound-healing therapies have been studied experimentally and clinically. Several studies have highlighted the impact of green medicine in relieving and healing wounds [16]. For this reason, researchers have paid close attention to essential oils (EOs) [17]. Their high volatility and lipophilicity, easy degradability, water insolubility, and sensitivity to environmental variables have limited their applications in the pharmacological field [18]. To overcome problems and difficulties, essential oils have been loaded into nanoparticles to prevent their degradation [19], improve and facilitate their penetration, increase their affinity for targets, and accelerate their accumulation process in different cell types [20].

As part of the search for a natural therapy to heal wounds, our contribution focuses on the choice of the genus *Pituranthos plant*. The genus *Pituranthos,* or *Deverra,* includes about 20 species [21]. It is associated with different uses in traditional medicine [22]. Some plants of this genus are traditionally used as a natural remedy to cure many illnesses and symptoms, such as common rheumatic disorders, diabetes, digestive problems, asthma, and hepatitis [22]. *Pituranthos tortuosus* (Coss.) Maire is the most common species; it belongs to the Apiaceae family and has recently been classified as *Deverra tortuosa* (Desf.) DC. It is a small perennial woody aromatic plant with a characteristic pungent or aromatic scent. It is widely grown in the Northern coastal areas of Africa [23], in central and southern Tunisia [24], in Libya [23], in Egypt [25], and in Saudi Arabia [26]. *P. tortuosus* is thoroughly rich in secondary metabolites such as terpenoids, steroids, flavonoids and glucosides [27], essential oils [28], lactones [28], esters [29], furocoumarins [29,30], and marmin [30]. 

Therefore, it has recently become of great interest to examine and discover the chemical composition of essential oils from aerial parts of *P. tortuosus,* which have shown interesting therapeutic potential and biological activities. The volatile fractions were extracted from the aerial parts, analyzed, and identified by gas chromatography–mass spectrometry, where 4-terpineol, dillapiole, sabinene, (*Z*)-3-butylidenephthalide, (*Z*)-ligustilide, *p*-cymene, and limonene dominated the results. Several studies report various properties for essential oils, being screened for their antimicrobial activity against Gram+ and Gram− bacteria [24,27,31], as well as their antifungal activity [22], anti-inflammatory activity [32], antioxidant activity [28,31], allelopathic potential [22], larvicidal activity, fumigant toxicity, and insecticidal activity [22,33]. 

In the field of medical technology, researchers are facing a major obstacle to accelerate the healing mechanism. A promising approach is needed to ensure the transport and simultaneous delivery of drugs to targeted areas and improve traditional and current treatments [34]. The increasing use of nanomaterials in wound healing is undeniable, and clinical studies based on nanotherapy are numerous [35]. The application of these nanostructures is an effective strategy to treat bacterial infections, develop new antibacterial agents that are able to inhibit the growth of pathogenic bacteria, and accelerate healing. They offer considerable advantages for targeting specific cells [36]. To optimize the potential of bioactive secondary metabolites, a nanoscale size reduction process is fundamental. New properties of nanostructures facilitate targeted drug delivery, tissue penetration, and cellular responses [37]. A nanoemulsion is a liquid–liquid dispersion with droplet sizes in the nanoscale, typically ranging from 20 to 200 nm [38]. It is a kinetically stable, clear dispersion of two immiscible phases, the oil phase and the water phase, in combination with surfactant molecules [39]. Nanoemulsions (NEs) have gained attention as vehicles for drug delivery due to their ability to improve the bioavailability and efficacy of loaded bioactive compounds [40]. NEs have been shown to have promising wound-healing properties [41], including antimicrobial and anti-inflammatory effects and the ability to promote cell proliferation and tissue regeneration [42]. The synthesis of nanoemulsions can be [43] achieved through various methods, including low-energy and high-energy approaches. In our study, the high-energy method was employed, utilizing a rotor–stator homogenizer operating at 13,000 rpm. The rotor–stator works by subjecting the liquid to high shear forces, which break down the oil phase into tiny droplets [44]. Our investigation is centered on evaluating the therapeutic potential of *Pituranthos tortuosus* essential oil (P. tort. EO) within a nanoemulsion–gel formulation designed for treating induced skin wounds in rats. The plant was harvested in Ben Guerdan, southeast Tunisia; between April and May, the essential oil was meticulously extracted from the aerial parts (leaves and tree stems). Significantly, no prior research has explored the combined effects of P. tort. EO within a nanoemulgel formulation for any specific medical indication. The acknowledged rich composition of P. tort. EO, featuring terpenoids such as sabinene, p-cymene, limonene, and dillapiole, renowned for their anti-inflammatory, antioxidant, and antimicrobial properties, leads us to hypothesize that these compounds could exert a positive impact on burns and wounds. Our study aims to pioneer the development and exploration of the nanoemulsion–gel formulation, offering innovative approaches to enhance the efficacy of wound-healing therapies and providing valuable insights into the potential of P. tort. EO in this context. 

## 2. Results and Discussion

### 2.1. Essential Oil Yield

The average extraction yield was 0.45%. Comparing our results with previous research, we observed that the obtained yields were higher (R = 0.45) than the reported value (0.25%) for *P. tortuosus* essential oil harvested during the flowering stage in the Monastir region (central Tunisia). A study showed that the EO yield is higher during the fruiting stage of *P. tortuosus* collected in northern Egypt (0.4%). 

The difference in yield as well as the difference in the chemotype (myrtenol and 4-terpineol) of two volatile fractions extracted from the aerial part of *P. tortuosus* growing in Tunisia in the regions of Monastir (central Tunisia) and Ben Guerdan (Southern Tunisia) can be attributed to the maturity levels of the plant species, climatic conditions, geographical situations (latitude, longitude, altitude, relative humidity, soil chemicals, and winds), method of extraction, duration, conditions of drying, and the management of the plants [22,45,46].

### 2.2. Chemical Composition of the Essential Oils

The essential oils from *P. tortuosus* were analyzed using GC-MS, and 38 compounds were identified (Table 1). Monoterpenes were the most abundant compounds (57.8%), followed by non-terpene derivatives (18.9%) and phenylpropanoids (13.4%). In particular, myrtenol, sabinene, limonene, *p*-cymene, 3-butylidenephthalide, and α-pinene were identified as the primary constituents of the EO collected from Mazdour, Governorate of Monastir, Center of Tunisia, in November and April. Similarly, sabinene, myrcene, α-pinene, *cis*-verbenol, *cis*-ocimene, *p*-cymene, α-terpinene, and *trans*-ocimene were found to be the major compounds in the EO obtained from fresh and dried herbs gathered in Beni-khedech, Medenine Southern Tunisia, respectively [33]. The chemical profile of the volatile oils gathered from Ben Guerdan, Medenine, South Tunisia, was distinct from that of other areas. The major compounds in the EO collected in the spring in Southern Sinai of Egypt were camphene, borneol, 1,8-cineole, α-pinene, and carvacrol. At the same time, dillapiole was found to be the main component of EO gathered from Egypt in another study [47].

### 2.3. Preparation and Characterization of the Nanoemulsion

**Figure 1:**
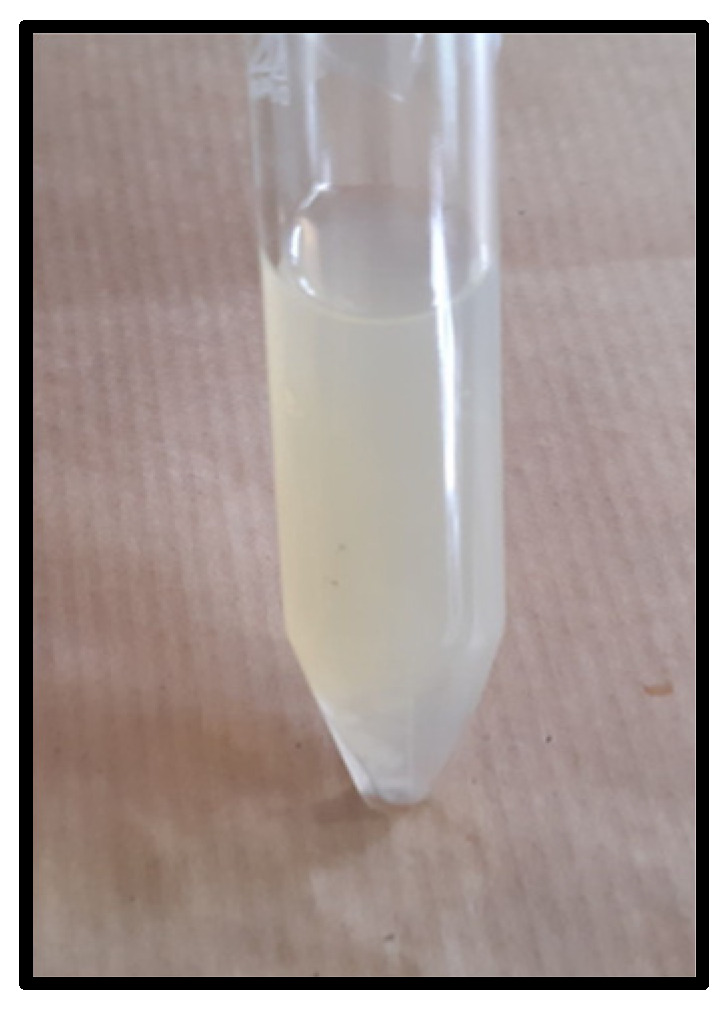
Prepared nanoemulsion.

The nanoemulsion containing EO was prepared successfully using the high-mechanical-energy method, as previously described. The obtained formulation was homogenous, with a translucent appearance, as illustrated in Figure 1. Particle size and zeta potential measurements were conducted to confirm the quality of the preparation. 

#### 2.3.1. Droplet Size Analysis and Distribution

Within the evaluation criteria for the nanoemulsion (NE), certain fundamental parameters significantly determine its quality. Among these, the particle size, measured at 27 ± 0.4 nm, and the polydispersity index (PDI) of 0.3 emerge as central indicators. These parameters are crucial in assessing the stability, uniformity, and overall efficacy of the NE formulation. Earlier findings have indicated that particles measuring below 300 nm have the potential to penetrate into deeper skin layers [50], thus affirming the suitability of our prepared NE for this intended use. Furthermore, the formulation’s PDI of 0.3 signifies excellent uniformity. 

#### 2.3.2. Zeta Potential

The polarity of emulsion droplets significantly influences the efficiency of emulsification. The increased electrostatic repulsive forces among nanodroplets effectively hinder coalescence within the resulting nanoemulsion. Conversely, reduced electrostatic repulsion can trigger phase separation, negatively impacting the formulation’s performance [15,17]. Comparative analyses of particles’ electrophoretic mobility offer valuable insights in this regard, commonly represented as zeta potential using the Smoluchowski equation. The prepared nanoemulsion demonstrated a zeta potential value of −22.8 ± 1.4 mV, indicating a negatively charged formulation with a favorable zeta potential [51]. These findings suggest the formulation’s stability due to its value and negative charge, emphasizing its potential efficacy. Conversely, several studies have outlined that the ability of nanoparticles to penetrate skin layers remains consistent irrespective of their charge [52,53]. Consequently, the developed nanoemulsion particles, despite their negative charge, are deemed highly suitable for skin delivery purposes.

#### 2.3.3. Thermodynamic Stability of the Optimal NE

The optimal NE, F7, showed no signs of instability, such as phase separation, coalescence, or creaming, and retained its characteristic bluish appearance after preparation. Stability is a significant advantage as it can help to preserve the therapeutic potential of phytochemicals [54]. Nanoemulsions, like F7, benefit from enhanced stability owing to their nanometric droplet size. It was demonstrated that this low size (typically less than 200 nm) offers kinetic stability, preventing gravitational separation and droplet aggregation [55]. Moreover, the small droplet size results in a larger interfacial area per unit volume, promoting stronger interaction between oil and water phases and thus enhancing stability [56]. 

**Figure 2:**
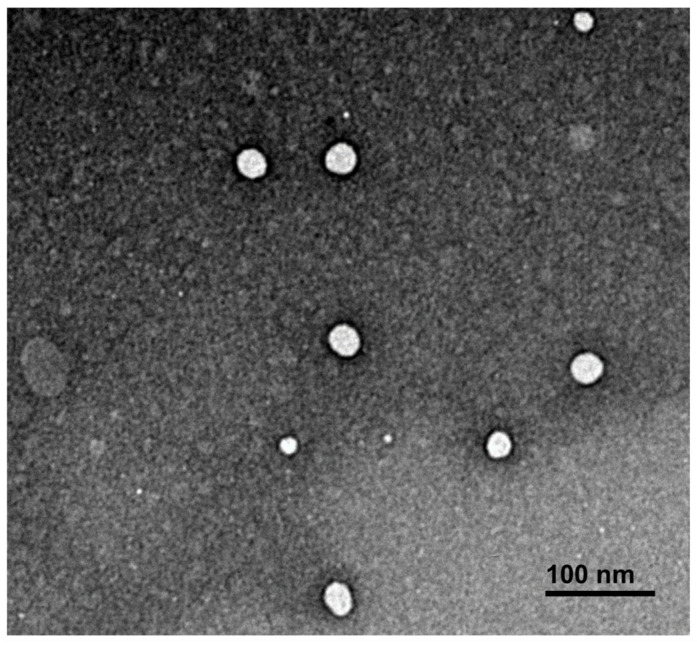
Optimal NE surface morphology using transmission electron microscopy.

TEM examinations were conducted to assess the surface morphology of the nanoemulsion, revealing well-dispersed spherical droplets within the nanometer size range, as represented in Figure 2. The observed stability and high efficacy of the formulation were evident through the droplets’ ability to resist the electronic beam, resulting in clear and distinct images. This resilience can be attributed to the presence of essential oil solubilized in triacetin, an oily vehicle that acts as a protective barrier against the degradation of the EO by the electronic beam. This finding aligns with the results obtained from other characterization tests, further underlining the quality and stability of our formulation [57]. Overall, these findings validate the success of our low-temperature nanoemulsification technique in achieving a stable and high-quality product.

### 2.4. Gelification and Characterization of the Optimal Nanoemulsion Gel (NE/Gel)

**Figure 3:**
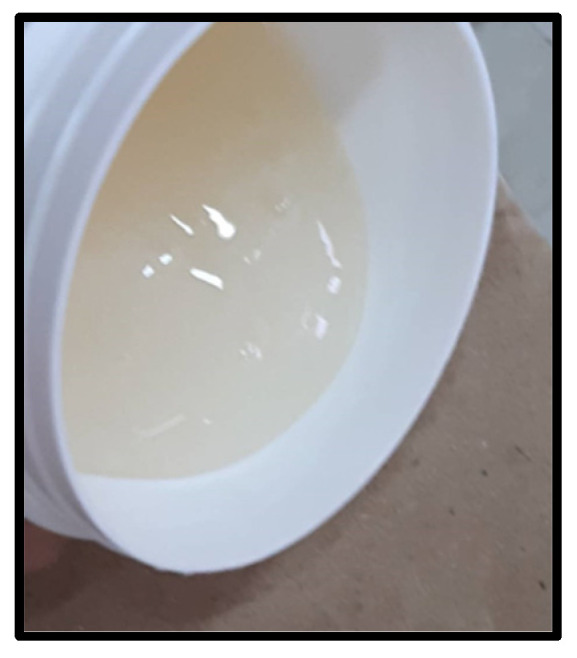
Nanoemulsion gel encapsulating *Pituranthos tortuosus*’s EO.

The gelification of the prepared NE is a critical process in topical treatments. The liquid NE is unsuitable for wound-healing treatments due to its low viscosity, which can result in the loss of the applied formulation and a subsequent lack of efficacy. However, by undergoing the gelification process, the NE viscosity increased, enhancing its adherence and making it more suitable for effective wound-healing treatments [58]. Sepimax Zen^®^ was utilized as the gelling agent at a concentration of 2.25%. The resulting NE/Gel, shown in Figure 3, exhibited a homogeneous texture with a non-adhesive touch. The efficacy of the gelification step can be attributed to the selection of the gelling agent. Sepimax Zen^®^, a non-ionic polymer renowned for its thickening, stabilizing, and texturizing properties, facilitated the creation of smooth and sophisticated gels with a velvety, translucent appearance.

#### 2.4.1. pH Measurement

In this study, the pH of the NE ranged from 6.0 to 6.3. After gelification, the pH of the formulation was within an acceptable range of 6.2 to 6.3. Therefore, the risk of skin irritation upon topical administration of the formulation is low. The pH can have an impact on its compatibility with the skin and cause potential irritation. It is important to maintain a pH similar to that of the skin, which is typically between 4.5 and 6.5 [59]. The presence of polyphenolic components with an acidic character in the EO could contribute to the formulation’s acidity. Additionally, the use of Sepimax Zen, which does not affect the pH of the preparation, may also explain the measured pH range.

#### 2.4.2. Rheology Study

**Figure 4:**
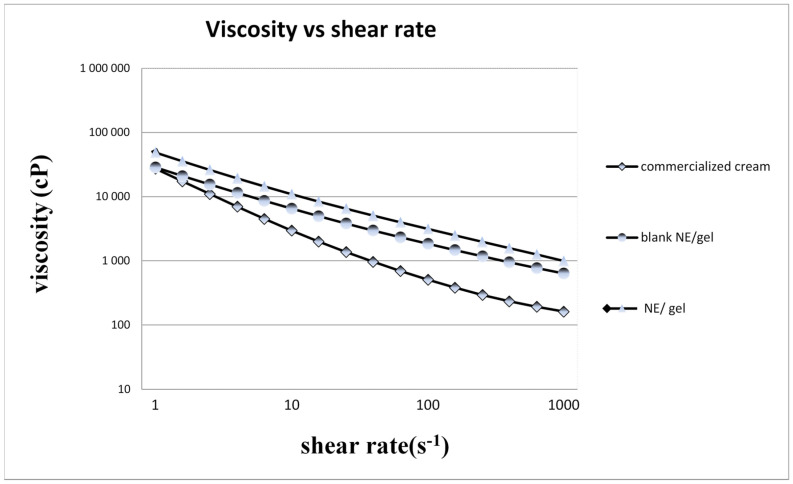
The rheological behavior of samples.

It is well known that the application and acceptance of topical formulations are highly dependent on the flow properties of the final product [60,61]. The rheological behavior of the investigated formulations is shown in Figure 4.

The formulations showed a decrease in viscosity with the increasing shear rate. These results can give interesting insights into the development of formulations for topical applications with the desired spreadability.

All formulations present shear-thinning (or pseudoplastic) behavior, and this means that the viscosity decreases as the shear rate increases. Pseudoplastic characteristics are of real interest for topical formulations [62].

The viscosity of the optimal NE/Gel is the highest among all samples, indicating that the gelification process significantly increased the consistency of the liquid NE.

Overall, the rheological properties of the samples suggest that the addition of essential oil and the gelification process have a significant impact on the consistency and texture of the NE. Our NE/Gel appears to have the most desirable consistency for topical application.

The rheological profile of a semi-solid product is a critical quality attribute [62], as it influences patient adherence, drug release, manufacturability, and product stability [63,64,65].

### 2.5. In Vivo Wound-Healing Study

**Figure 5:**
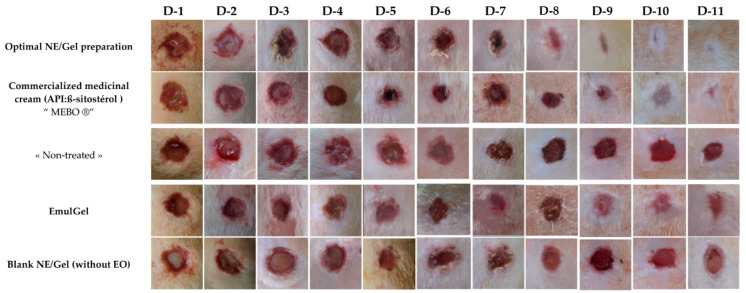
Different phases of in vivo healing.

**Figure 6:**
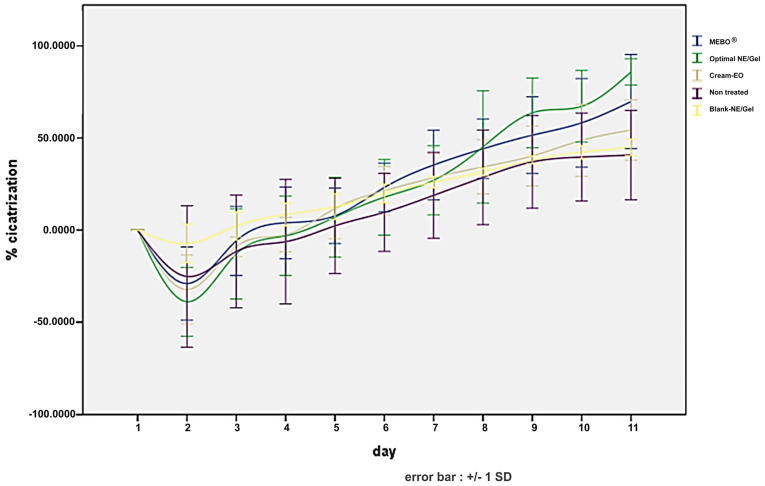
Healing kinetics.

The present study examines the effects of *P. tortuosus* EO on wound healing. The results of the experiment are summarized in Figure 5 and Figure 6. Many formulations were tested, as listed in Table 3.

During the 11 days of observation, the untreated wounds progressed very slowly towards healing. Acute signs of inflammation, with the presence of exudate, were observed on days 2 and 3, which is normal during the healing process but is an indicator of chronicity. Similar observations were made for wounds treated with blank NE/Gel. The rats showed suppuration, and the progression towards healing was very slow for wounds treated with the blank NE/Gel, confirming that none of the components, other than EO, used in this preparation affect wound healing.

In contrast, wounds treated with NE/Gel showed rapid improvement within 9 days, with a mean percentage of wound healing reaching 85.87%. Notably, the application of the preparation resulted in the formation of slightly dark crusts by day 5, which gradually detached starting from day 7, leading to accelerated wound closure and complete healing by the 10th day. Conversely, the conventional Emul/Gel demonstrated a slower healing trajectory compared to the NE/Gel, albeit with the onset of blackish crusts from day 9 onwards, with a mean percentage of wound healing of 54.31%.

Statistical analysis further supports these observations, revealing a significant difference in wound-healing percentages between the NE/Gel and the emulgel as well as between the NE/Gel and untreated or blank NE/Gel-treated wounds (*p* < 0.05). Several studies have emphasized the diversity of biological activities and therapeutic properties of *P. tortuosus* [22,27,45,47]. However, no preliminary tests for the wound-healing ability of this species are present in the literature.

Essential oils, including *Pithurantus* EO, are fundamental to wound healing due to their complex chemical composition [27,28]. Rich in hydrocarbons and oxygenated mono-terpenes, the volatile fraction of this extract substantiates its therapeutic effects [27,28]. 

Terpenoids such as sabinene [66] (8.7%), *p*-cymene [67,68] (6%), limonene [69,70] (5.2%), and dillapiole [66] (13%) play crucial roles, offering anti-inflammatory, antioxidant, and antimicrobial potential [32]. 

This study suggests the promising potential of nanoemulsion (NE) formulations incorporating *P. tortuosus* EO in wound-healing therapies. Miahi et al. have indicated that nanodroplets, owing to their significantly high surface-to-volume ratio, may potentially modify physicochemical properties, thereby influencing the pharmacokinetics and pharmacodynamics of active compounds [36]. Moreover, findings by Souto et al. demonstrate that the characteristic Brownian motion of NEs may promote diffusion through skin layers, facilitating deeper penetration and enhanced therapeutic delivery [71]. Additionally, as reported by Algahtani et al., the negatively charged nanodroplets could help to concentrate active compounds locally while minimizing systemic leakage, which is detrimental in wound-healing applications [72]. These insights collectively suggest the promising role of NE formulations in potentially expediting wound-healing processes.

The effectiveness of the nanoemulsion gel (NE/Gel) containing the EO of *P. tortuosus* in promoting wound healing has been demonstrated. The distinct benefit of employing the NE/Gel in this study lies in its hybrid nature, combining both nanoemulsion and gel characteristics. This unique combination facilitates the solubilization of lipophilic essential oils within the nanoemulsion domains. Additionally, its high aqueous content contributes to enhanced spreadability on the skin [73].

Moreover, the gel form ensures prolonged residence over the skin, hence allowing a better chance for skin penetration and enhanced therapeutic effects.

As evidenced in a recent study conducted by Alyoussef et al. (2021), the utilization of curcumin and resveratrol loaded in a nanoemulsion–gel exhibited superior wound contraction rates compared to gels and emulgels. This highlights the promising potential of nanoemulgels as an effective topical delivery system for wound healing [74]. Hence, the objective of this study was to assess the impact of a nanoemulsion–gel formulation containing *Pituranthos tortuosus* essential oil on the skin-healing process of induced wounds.

## 3. Conclusions

This work represents a significant contribution to enhancing the healing potential of the essential oil extracted from the aerial part of Pituranthos tortuosus, a plant native to Tunisia. The prepared nanoemulsion–gel demonstrated remarkable efficacy in accelerating the healing process. Notably, its therapeutic effects were observed after only 10 days, surpassing those of commercially available healing creams. This nanoemulsion-based preparation offers a promising strategy for managing the healing process and is very promising for the treatment of various lesions and skin disorders in vivo. In perspective, our work opens the door to promising future directions. Future studies may include identifying specific markers for wound-healing mechanisms, exploring the cellular-level interactions of the nanoemulsion, conducting human trials to validate its efficacy, and promoting collaborative efforts in various medical applications. These paths highlight the prospective importance of our research and its contribution to the advancement of essential oil-based therapies.

## 4. Materials and Methods

Chemicals: Tween^®^ 80 (HLB = 15) and Span^®^ 80 (HLB = 4.3) were obtained from Sigma-Aldrich (Tunis, Tunisia); SEPIMAX ZEN ™ was obtained from SEPPIC (Paris, France); and Triacetin 1,-2,-3-propanetriol triacetate, paraffin oil (P.O), and ethanol were obtained from Fluka Chemie (Buchs, Switzerland). 

### 4.1. Plant Material and Extraction of Essential Oil from P. tortuousus

*P. tortuosus* plants were harvested in Ben Guerdan, south-eastern Tunisia, between April and early May 2019. The plant aerial parts (leaves and tree stems) were collected in a clean cloth bag, dried away from light and moisture for 20 days, and then cut into small pieces. Initial visual identification and classification occurred on-site during plant collection in Ben Guerdan. Subsequent verification and confirmation were diligently performed at the Drug Development Laboratory (University of Monastir), ensuring precise botanical characterization.

The extraction method involved hydrodistillation, where seventy grams of plant material was processed for four hours using a Clevenger apparatus, following the procedure outlined by Umesh et al. [75]. The essential oil (EO) was collected and stored at 4 °C in a glass bottle with an airtight cap.

### 4.2. GC-MS Analysis of the EO

Gas chromatography–electron impact mass spectrometry (GC–EIMS) analyses were performed with an Agilent 7890B gas chromatograph (Agilent Technologies Inc., Santa Clara, CA, USA) equipped with an Agilent DB-5MS (Agilent Technologies Inc., Santa Clara, CA, USA) capillary column (30 m × 0.25 mm; coating thickness 0.25 μm) and an Agilent 5977B single quadrupole mass detector (Agilent Technologies Inc., Santa Clara, CA, USA). The analytical conditions were as follows: injector and transfer line temperatures: 220 and 240 °C, respectively; oven temperature programmed from 60 to 240 °C at 3 °C/min; carrier gas helium at 1 mL/min; injection of 1 μL (5% HPLC-grade *n*-hexane solution); split ratio: 1:25. The acquisition parameters were as follows: full scan; scan range: 30–300 *m*/*z*; scan time: 1.0 s. The constituents were identified using computer matching against commercial (NIST 14 and ADAMS) and homemade library mass spectra built up from pure substances and components of known oils and MS literature data, as well as by comparison of the linear retention indices of the constituents relative to the series of *n*-hydrocarbons and a comparison of their retention times with those of authentic samples [48,76].

### 4.3. Nanoemulsion Preparation (NE)

A nanoemulsion loading *Pituranthos tortuosus* EO was formulated following a high-mechanical-energy method, which we reported in our previous publication [77]. 

Preliminary studies and the team’s expertise were both employed to meticulously determine the precise amounts and ratios of excipients in the formulations. The compositions of the EO-loaded NE and the blank NE are listed in Table 2. 

Briefly, the formulation involved the preparation of two distinct phases: The oily phase consisted of *Pituranthos tortuosus* essential oil (EO), an oily vehicle (triacetine), the co-surfactant (Span 80), and a co-solvent (ethanol). The aqueous phase comprised a surfactant (Tween 80) and deionized water (aqueous vehicle). 

Then, the two phases were homogenized for 2 min at 13,000 rpm using a POLYTRON rotor–stator homogenizer (KINEMATIKA, Lucerne, Switzerland) equipped with the “G—GAS TIGHT” (KINEMATIKA, Lucerne, Switzerland) aggregate with integrated mechanical seals. It is characterized by a diameter of 20 mm, a dispersion axis length of 210 mm, and processing volume ranging from 100 mL to 2000 mL. The nanoemulsion was prepared at room temperature, and strict temperature control measures were implemented during the process to ensure it did not exceed 35 °C. The prepared formulations were conserved for 24 h at room temperature before their characterization.

### 4.4. Characterization of the Nanoemulsion

#### 4.4.1. Particle Size Distribution and Polydispersity Index Determinations 

The particle size and polydispersity index (PDI) of all formulations were determined at 25 °C by DLS (dynamic light scattering) using the Zetasizer Nano-S laser particle size analyzer (Malvern Instruments, Worcestershire, UK) with a measurement range of 0.3 nm to 10 µm. DLS is a technique used to measure the size of particles, including nanoparticles, in a solution. It works by analyzing the random changes in the intensity of light scattered from a suspension or solution [78]. All measurements were performed in triplicate.

#### 4.4.2. Zeta Potential Measurement

The zeta potential was determined by a Malvern Zetasizer Nano-Z type apparatus (ZEN 3600) (Malvern Instruments Ltd., Malvern, Worcestershire, UK). The analysis was repeated 3 times.

#### 4.4.3. Thermodynamic Stability Study

The prepared NE was centrifuged at 3500 rpm for 15 min to check for signs of instability, such as creaming or coalescence, and to measure the droplet size. Additionally, three samples of nanoemulsions were subjected to six cycles between refrigerator temperature (4 °C) and 40 °C with a storage period of 48 h and were visually inspected after centrifugation for signs of instability, including phase separation, coalescence, or creaming [79].

#### 4.4.4. Transmission Electron Microscopy Study

The morphology of the NE was performed by a JEM-100S Electron Microscope (JEOL Ltd., Tokyo, Japan). The magnification and diffraction modes were used to reveal the shape and size of the NE. The diluted NE (1/10) was deposited on a holey film grid and observed after drying. The grid underwent negative staining using a 2% uranyl acetate solution. This technique was employed to enhance the visibility of light elements (such as C, O, H, N) in water-dispersed samples during transmission electron microscopy (TEM) observations. The introduction of a uranium salt solution created a surrounding medium for hydrophobic nanoemulsion droplets, exploiting the high electron density of uranium to produce a contrasting effect. Consequently, the structures of interest appeared bright against a dark background.

### 4.5. Emulsion Preparation and Gelification

An emulsion with the same composition as the nanoemulsion (NE) was prepared using a propeller stirrer at 700 rpm for 5 min. Gelification of the emulsion was achieved using 2.25% Sepimax Zen^®^ as a gelling agent (INCI: polyacrylate crosspolymer-6). The Sepimax ZEN ™ was weighed, sieved, and added by a small fraction to the emulsion. Moderate stirring was sustained until achieving a gel with the desired viscosity The preparation of the emulsion–gel was conducted at room temperature. This gelification process resulted in an emulgel, which will be compared with the gelified nanoemulsion in the in vivo wound-healing study.

### 4.6. Gelification Process and NE/Gel Characterization

The NE was gelified using 2.25% Sepimax Zen^®^. The preparation of the nanoemulsion–gel (NE/Gel) was carried out at room temperature. The Sepimax ZEN™ was weighed, sieved, and added by a small fraction to the NE. Moderate stirring was sustained until achieving a gel with the desired viscosity. In addition to the NE/Gel, the same protocol of gelification was applied for a conventional emulsion encapsulating the essential oil. 

#### 4.6.1. pH Measurement

The pH of the NE/Gel was measured using a pH meter in triplicate and then averaged. The formulation should have a pH that aligns with the inherent acidic pH of the skin, ensuring compatibility for optimal efficacy and inhibiting the development of bacterial infections on the skin surface. 

#### 4.6.2. Rheological Study

The rheological behaviors of the optimal nanoemulgel, a commercialized wound-healing cream, and the blank nanoemulgel were studied using a rotational rheometer, the Malvern^®^ kinexus plate cartridge (Worcestershire, UK), equipped with a cone plate (geometry PU40 SR3300SS) which enables measurements with a sample and a constant shear rate on the entire surface of the cone. The study was performed 24 h after sample preparation and storage at room temperature. The flow behavior was studied by continuous shear investigations, performed to evaluate the viscosity (cP) as a function of the shear rate (s1). The study started with a shear rate of 1 s^−1^ up to 1000 s^−1^. A minimum rest period of 1 min was applied between each section. Measurements were conducted at 25 °C by performing 5 repetitions. The data were recorded and processed using the rSpace software interface version 2.0 [80].

### 4.7. In Vivo Wound-Healing Study

Five groups of male Wistar rats were used, as described in Table 3. The animals’ average mass was around 250 ± 20 g. They were kept in a conventional laboratory setting with 12 h light/dark cycles, a temperature of about +25 °C, and a standardized meal of water and granules designed for the diet of mice. Each intervention involved careful handling of the animals according to the PREPARE reference guide’s instruction (“Planning Research and Experimental Procedures on Animals: Recommendations for Excellence”) A3 to protect animal welfare, minimize unnecessary suffering, and avoid any negative impacts that could affect the experiment outcomes. 

**Figure 7:**
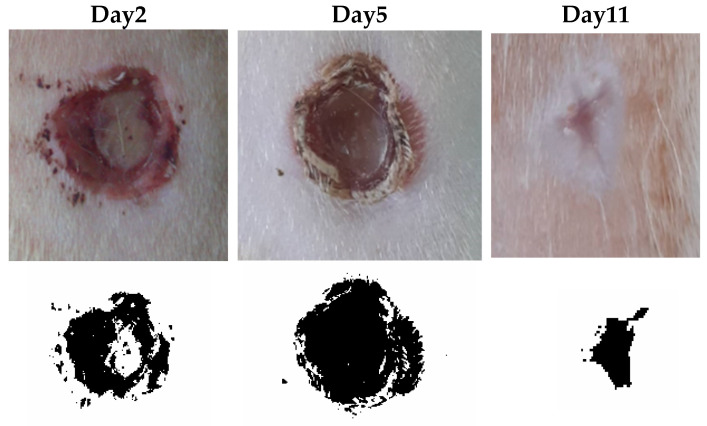
Wound-healing rate (WHR).

The areas of the wounds were measured using photography combined with ImageJ software version 1.38. The wounds were photographed with a ruler to allow for a subsequent calibration in ImageJ, as shown in Figure 7.

The backs of the anesthetized rats were first shaved with a PRITECH^®^ electric trimmer (CDiscount, Paris, France), and the skin was then thoroughly cleaned and disinfected by rinsing with ether and alcohol.

Three circular incisions (7 mm in diameter) were made on each rat’s back using a sterilized chisel and a stainless steel delimiter. Daily observations of the lesions were made to track how they were changing over time. Then, fresh compresses and bandages were placed on top of them. Before each treatment, the rats were put to sleep and photographed once a day at the same time for 11 days; the therapy was administered to the whole lesion in a rich layer about 2 mm thick. The animals were divided into 5 groups as indicated in Table 3, with 4 rats in each group, following the protocol described by Algahtani et al. with slight modifications [72].

The image determined the wound outline, and then the ImageJ software (version 1.38) calculated the wound area (Image-J, U.S. National Institutes of Health). The WHR (wound-healing rate) was calculated according to the following formula: WHR = initial area−final area/initial area [81].

### 4.8. Statistical Analysis

All statistical analyses were conducted using the SPSS statistical software (version 24, IBM Corp., Armonk, NY, USA). Data are presented as the mean ± standard deviation (SD). Pairwise comparisons between specific treatment groups were performed using independent samples *t*-tests. The significance level for all statistical tests was set at *p* < 0.05.

## Figures and Tables

**Table 1:** Chemical composition of the essential oil from *P. tortuosus*.

N°	Compounds	* LRI	Lit LRI	** *P. tortuos* EO ± STDEVA	N°	Compounds	Lit LRI	* LRI	** *P. tortuosus* EO ± STDEVA
1	α-Thujene	933	930	0.80 ± 0.06	24	Bornyl acetate	1289	1287	0.80 ± 0.10
2	α-Pinene	941	939	2.10 ± 0.15	25	*p*-Cymen-7-ol (syn. cumin alcohol)	1291	1290	0.90 ± 0.21
3	**Sabinene**	**977**	**975**	**8.70 ± 0.81**	26	Carvacrol	1299	1299	1.70 ± 0.10
4	β-Pinene	982	980	0.60 ± 0.06	27	Pinanediol	1320	1317	0.40 ± 0.12
5	Myrcene	992	991	0.60 ± 0.06	28	*p*-Mentha-1,4-dien-7-ol	1330	1331	0.40 ± 0.06
6	α-Phellandrene	1006	1003	0.30 ± 0.06	29	α-Longipinene	1334	1338	0.90 ± 0.15
7	α-Terpinene	1020	1017	0.90 ± 0.06	30	Methyl eugenol	1404	1403	0.40 ± 0.06
8	***p*-Cymene**	**1028**	**1026**	**6.00 ± 0.26**	31	β-Bisabolene	1506	1508	0.50 ± 0.06
9	**Limonene**	**1032**	**1029**	**5.20 ± 0.38**	32	Spathulenol	1578	1576	0.70 ± 0.06
10	γ-Terpinene	1063	1060	2.50 ± 0.15	33	**Dillapiole**	**1621**	**1623**	**13.00 ± 0.62**
11	Terpinolene	1090	1089	1.10 ± 0.06	34	β-Eudesmol	1651	1650	1.00 ± 0.12
12	*cis*-*p*-Menth- 2-en-1-ol	1123	1122	1.80 ± 0.10	35	**(*Z*)-3-Butylidenephthalide**	**1673**	**1677**	**8.50 ± 0.64**
13	α-Campholenal	1125	1226	0.40 ± 0.06	36	(*E*)-3-Butylidenephthalide	1718	1716	3.90 ± 0.47
14	*trans*-*p*-Menth- 2-en-1-ol	1142	1141	0.90 ± 0.06	37	**(*Z*)-Ligustilide**	**1741**	**1737**	**6.40 ± 0.62**
15	Camphor	1145	1146	0.40 ± 0.06	38	Hexahydrofarnesylacetone	1845	1845	0.70 ± 0.10
16	Sabinaketone	1159	1159	0.80 ± 0.06		**Monoterpene hydrocarbons**	**28.90 ± 1.97**		
17	**4-Terpineol**	**1179**	**1177**	**16.20 ± 1.46**		**Oxygenated monoterpenes**	**28.90 ± 1.37**		
18	*p*-Cymen-8-ol	1185	1183	1.40 ± 0.06		**Sesquiterpene hydrocarbons**	**1.40 ± 0.20**		
19	α-Terpineol	1191	1189	1.10 ± 0.10		**Oxygenated sesquiterpenes**	**1.60 ± 0.15**		
20	*p*-Mentha-1,5- dien-7-ol	1193	1194	0.30 ± 0.00		**Phenylpropanoids**	**13.40 ± 0.68**		
21	Myrtenal	1194	1196	0.50 ± 0.06		**Apocarotenes**	**0.70 ± 0.10**		
22	Carvone	1244	1243	0.50 ± 0.00		**Non-terpene derivatives**	**18.90 ± 1.74**		
23	*trans*-Ascaridol glicol	1271	1269	0.40 ± 0.06		**Total identified**	**93.80 ± 0.51**		

* LRI: Linear retention indices; ** *n* = 3. Compound numbers in red are from reference [48], numbers in blue are from reference [46], and numbers in green are from reference [49]. Bold indicates the main compounds.

**Table 2:** Composition of NE formulae.

Composition (*w*/*w*)	Nanoemulsion (NE)	Blank NE
EO %	1	0
Triacetin %	10	10
Span 80%	5.6	5.6
Ethanol %	1	1
Tween 80%	14.4	14.4
Deionized water %	68	69

**Table 3:** Tested formulations and corresponding rat group.

Group	Titer
G1	NE/Gel preparation
G2	Blank NE/Gel (without EO)
G3	Conventional Emulsion/Gel “EmulGel”
G4	Non treated
G5	Commercialized medicinal cream (API: ß-sitostérol) “MEBO ^®^”

## Data Availability

The raw data supporting the conclusions of this article will be made available by the authors on request.
