# Correction: Bahloul et al. Investigating the Wound-Healing Potential of a Nanoemulsion–Gel Formulation of *Pituranthos tortuosus* Essential Oil. *Gels* 2024, *10*, 155

**DOI:** 10.3390/gels12050414

**Published:** 2026-05-09

**Authors:** Badr Bahloul, Enis Ben Bnina, Assia Hamdi, Luis Castillo Henríquez, Dhaou Baccar, Nesrine Kalboussi, Aïmen Abbassi, Nathalie Mignet, Guido Flamini, José Roberto Vega-Baudrit

**Affiliations:** 1 Drug Development Laboratory LR12ES09, Faculty of Pharmacy, University of Monastir, Monastir 5000, Tunisia; 2 LR21AGR03-Production and Protection for a Sustainable Horticulture (2PHD), Regional Research Centre on Horticulture and Organic Agriculture, IRESA, University of Sousse, Chott Mariem 4042, Tunisia; 3 Chemical and Biological Technologies for Health Group (UTCBS), Université Paris Cité, 75006 Paris, France; 4 Research Unit “Natural Bioactive Substances and Biotechnology” UR17ES49, Pharmacognosy Laboratory, College of Pharmacy of Monastir, University of Monastir, Monastir 5000, Tunisia; 5 Dipartimento di Farmacia, University of Pisa, Via Bonanno 6, 56126 Pisa, Italy; 6 National Nanotechnology Laboratory (LANOTEC), National Center for High Technology (CeNAT), San José 1174-1200, Costa Rica

In the original publication [1], there was a mistake in Figure 5 as published. Some images were unintentionally repeated during figure preparation and have now been replaced by images from additional experimental replicates corresponding to the same treatment days. The corrected Figure 5 appears below. The authors state that the scientific conclusions are unaffected. This correction was approved by the Academic Editor. The original publication has also been updated.


